# Antimicrobial Resistance as a Global Health Threat: The Need to Learn Lessons from the COVID‐19 Pandemic

**DOI:** 10.1111/1758-5899.13049

**Published:** 2022-03-16

**Authors:** Anishka Cameron, Regina Esiovwa, John Connolly, Andrew Hursthouse, Fiona Henriquez

**Affiliations:** ^1^ University of the West of Scotland Paisley UK

## Abstract

The global COVID‐19 pandemic has exacerbated existing health, social, and economic challenges and threatened progress towards achieving the UN sustainable development goals. We discuss lessons learned from the COVID‐19 pandemic for global policymaking for health security governance, with a particular focus on antimicrobial resistance. We identify One Health as the primary foundation of public health risk management owing to the collaborative, multidisciplinary, and multisectoral efforts that underpin the One Health approach and that enhance understanding of the complex interactions at the human–animal–environment interface. We discuss the narrow human‐centric focus of the One Health approach, highlight the underrepresentation of the environmental sector in One Health networks, and encourage greater representation from the environmental sector. Furthermore, we highlight the importance of the social sciences for health security research and the need for effective communication and trust. Finally, we underscore the importance of strengthened and collaborative health, social care, and disaster management systems. The application of these lessons will facilitate holistic, multisectoral, collaborative, and ethical actions on antimicrobial resistance.

**Policy Implications**

- The role of the environment in disease development and dissemination cannot be overlooked and this is key to tackling public health threats such as antimicrobial resistance (AMR) and COVID‐19.
- The COVID‐19 pandemic response has shown that risk communication that is timely, consistent, transparent, and from a trusted source, is crucial to public engagement and acceptance.
- The pandemic has dominated global health research and disrupted responses to global threats that intersect with it, as limited available resources throw up the challenge of balancing immediate COVID‐19 response needs with those of other concurrently occurring crises.
- Global policy‐makers need to strengthen health systems; create strong public health campaigns that demonstrate the contribution of human behaviours to the development of AMR; and establish resilient AMR surveillance programmes that estimate AMR burden.
- There are opportunities for AMR research to capitalise on the recent acknowledgement of the critical importance of the social sciences as a result of the experiences of the COVID‐19 pandemic.

## 1 AMR AS A ONE HEALTH PROBLEM WITH PANDEMIC POTENTIAL

This paper is a critical policy appraisal, informed by a systemic rapid review methodology, which details the lessons for those responsible for antimicrobial resistance (AMR) prevention and response, based on the experience of the COVID‐19 pandemic. There are several policy and governance‐focused analyses of the pandemic and each, in its own way, highlight the need for policymakers to avoid similar crises happening again (Cairney & Wellstead, 2020; Mazey & Richardson, 2020; Weible et al., 2020). Governance responses to COVID‐19 are analysed in this article to identify potential courses of action that should be applied by policymakers and governments with regards to the future of global health governance for AMR. This article also analyses the placement of AMR on the global health agenda as a result of the COVID‐19 pandemic and provides recommendations to avoid deprioritisation of coexisting health threats. Mazey & Richardson maintain that ‘COVID‐19 might possibly turn out to have been a seismic event in the process by which public policies are made’ (Mazey & Richardson, 2020). Moreover, Boin et al. (2021) also note that ‘crises are great teachers for those who are willing to learn’.

The COVID‐19 pandemic is not the world's first significant global health threat. In the past two decades alone, several emerging infectious disease outbreaks have surfaced, including previous coronaviruses (e.g. severe acute respiratory syndrome [SARS] and Middle East respiratory syndrome [MERS]), Ebola, avian flu, and Zika virus among others (WHO, 2021e). Beyond the threat of global infectious disease spread, a slower burning health crisis with pandemic potential has been evolving (Devlin, 2020). The rise in AMR is of long‐term global health concern, threatening an antimicrobial crisis in which microbials no longer respond to treatment, rendering even routine surgeries and common infections dangerous (O’Neill, 2016). Furthermore, the risk of AMR can complicate the transmission of zoonotic pathogens (infectious disease originating in animals), enabling the spread of resistant variants (Kim, 2017). Antimicrobial resistance causes approximately 700,000 deaths across the world on a yearly basis (O’Neill, 2016). The fatalities and direct costs caused by AMR could be substantially higher than for the COVID‐19 pandemic in the longer term. If left unaddressed, the cost of AMR could amount to $100 trillion by 2050 and lead to 10 million deaths per year (O’Neill, 2016). Research indicates that prescribing practices for addressing COVID‐19 may be accelerating AMR (Clancy et al., 2020). Antibiotic prescribing rates have risen during the pandemic, with almost 75% of COVID‐19 patients receiving antibiotics even in the absence of confirmed bacterial coinfections (Langford et al., 2021). As the misuse and overuse of antimicrobials is a well‐documented driver of AMR, this practice raises concerns over the contribution of unnecessary antibiotic use in COVID‐19 patients to future AMR (Hsu, 2020). This risk may be offset by the introduction of physical distancing and increased infection control practices, which may reduce opportunities for the spread of resistant microbes (Collignon & Beggs, 2020). However, the environmental impact of increased use of hand sanitisers and disinfectants, which contain antimicrobial (biocide) materials, is yet to be determined (Rezasoltani et al., 2020).

Aside from these concerns, the COVID‐19 pandemic has the potential to reframe the future of global governance for AMR if lessons translate into policy change. Notably, the emergence and spread of COVID‐19 has reinforced the need to better understand that the health of humans is inextricably linked to the health of animals and the shared environment they occupy. One Health acknowledges the relationship of human–animal–environment health in addressing health threats (One Health Global Network, 2015). The approach supports increased communication and knowledge integration between different sectors to mitigate health risks (Connolly, 2020). Implementation of a successful One Health approach involves perspectives beyond the natural sciences alone. It requires input from social scientists to aid understanding; facilitate communication between scientists, policymakers, and public; and ensure that the adopted strategies balance the interests and values of all stakeholders (Woldehanna & Zimicki, 2015). Applying this interdisciplinary approach to health enhances the surveillance, monitoring and evaluation, data sharing, and facilitates collective action (One Health Commission, 2021).

With this in mind, AMR is a quintessential One Health issue, with strong connections to each domain at the human–animal–environment interface (Robinson et al., 2016). Resistance can be found in humans, animals, and the environment, and can spread between species and locations (Rousham et al., 2018). Therefore, addressing the threat of AMR requires a holistic and collaborative effort to ensure that efforts in one domain are not weakened by neglect in another. To achieve this, the One Health approach needs to be reviewed to identify and overcome any barriers to the collaboration and inclusion of all relevant stakeholders and sectors. Global health threats require global solutions based on lessons learned; collaborative efforts to effectively manage the COVID‐19 pandemic and other global health threats, such as AMR, are critical. The actual lessons to be drawn from the COVID‐19 crisis are not simply about understanding how to prevent COVID‐19 rampaging across the nations of the world again. Rather, it is also about how the pandemic could present lessons to address other threats to humankind. Crisis management scholarship does, however, demonstrate that post‐crisis evaluations led by governments (through inquiry processes, for example) can be heavily politicised and there is no guarantee that lessons learned from crises or fiascos translate into actual policy and organisational change (Connolly, 2016; McConnell, 2011; Stark, 2018). Yet, it is a *moral endeavour* for academic researchers to make inroads into understanding how society can capitalise on the negative experiences of crises to foster a better future as far as possible.

The article is structured as follows. We discuss lessons from the COVID‐19 pandemic that would improve preparedness and response to other global crises, specifically AMR. The first lesson focuses on the need for increased environmental representation in One Health approaches. The second lesson highlights the essential role of timely, clear, and consistent risk communication in public understanding of infection prevention and transmission. The third lesson reflects on the deprioritisation of AMR activities during the COVID‐19 pandemic and suggests measures to address this. The fourth lesson points to the need for collaborative governance in our increasingly interconnected world. We emphasise the need for strengthened collaborative capacities for managing borderless public health threats, such as COVID‐19 and AMR. Finally, the article underlines the need for perspectives from the social sciences in bringing the above lessons together. This lesson focuses on understanding and navigating complex relationships to achieve buy‐in from stakeholders and communities towards the mitigation of AMR.

## 2 METHODOLOGY

In this critical policy appraisal, we applied a systematic rapid review methodology to identify lessons to be learned from the COVID‐19 pandemic in relation to managing and mitigating global health threats, specifically AMR. The method used was a multiple database search, including Ovid, PubMed, Embase, Web of Science, and Google Scholar, searched for primary and secondary sources that highlighted parallel issues between COVID‐19 and AMR. This was carried out in terms of the global health risk each present and the role of anthropogenic influences in their development and spread. Specific themes in COVID‐19 literature were distilled to those synchronised or aligned with AMR. These themes were subsequently used in the systematic rapid search carried out on Ovid and PubMed databases. The search strategy applied in the systematic search included terms from the prior identified themes, that is, *COVID‐19, health security, crisis management, AMR, antimicrobial resistance, collaboration, risk communication, One Health, pandemic, and social science*, used alone and in multiple combinations. Reference lists of relevant articles and grey literature searches were also conducted. The search was conducted between January and March 2021, and restricted to the English language. In all, 341 records were identified and duplicate records were removed. The records were searched to identify the different responses to the COVID‐19 pandemic, to understand the parallels between COVID‐19 and AMR, and to highlight lessons from the COVID‐19 pandemic that could be applied to the mitigation of AMR.

## 3 THE IMPORTANCE OF A ONE HEALTH APPROACH TO GLOBAL HEALTH SECURITY POLICY

The COVID‐19 pandemic is an example of a real consequence of neglecting the relationship between the health of humans, animals, and the environment (Dhama et al., 2020). The question now is whether enough has been done to achieve One Health in public health risk management, and how to improve current approaches for a sustainable future. The One Health concept is not new, but in the past decade, it has become increasingly recognised as an approach to understanding and mitigating public health threats at the human–animal–environment interface (Overgaauw et al., 2020). Although there is no single agreed definition of One Health, a common foundation to this approach exists. The One Health approach promotes the health of humans, animals, and the shared environment through collaboration across multiple disciplines and sectors at local, national, and global levels (Barton Behravesh, 2019). One Health facilitates such collaborative efforts through increasing awareness of the relationship between the health of humans, animals, and our shared environment. Applying a One Health approach to public health can stimulate innovation in research and strengthen policymaking through knowledge integration and systems thinking (Hitziger et al., 2018). Engaging with multiple disciplines, sectors, and stakeholders improves understanding of the complex interactions at the human–animal–environment interface in disease emergence and progression, and enhances the appreciation of the values and responsibilities of each actor involved (Kanankege et al., 2020). These collaborative capacities also allow for increased surveillance through data sharing and analysis, which translates to earlier detection, reduced disease risks, and cost‐saving benefits in the longer term (World Bank, 2012).

### 3.1 Sectoral representation in the One Health approach

**FIGURE 1:**
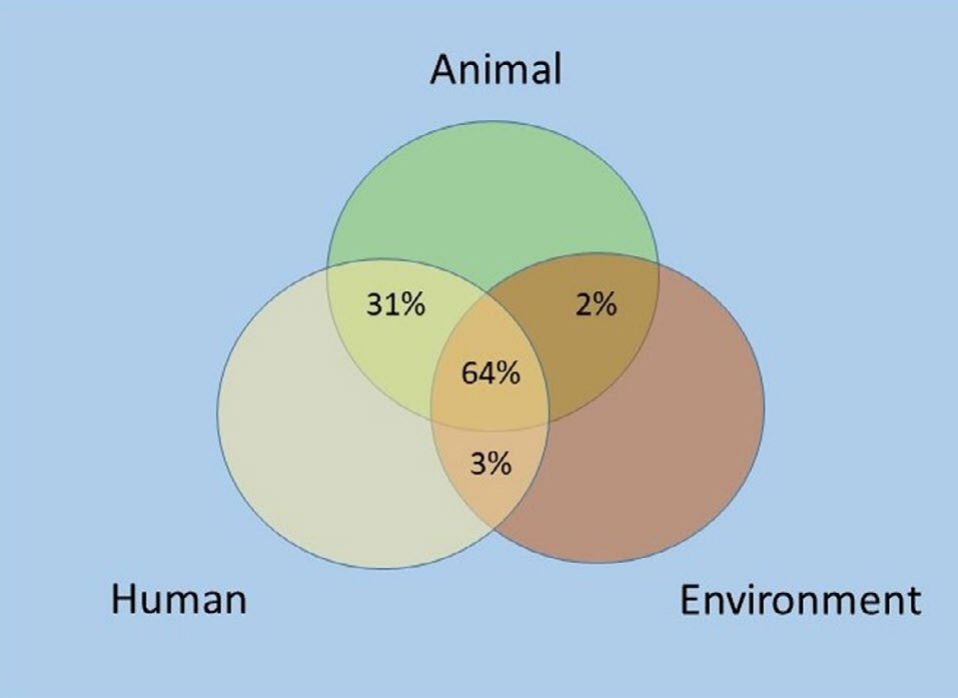
Systematic analysis of 100 One Health networks showing greater disengagement with the environmental sector (2018; created from Khan et al., 2018; not drawn to scale)

Although COVID‐19 has increased awareness of the importance of One Health, this does not eliminate pre‐existing challenges to its successful implementation. Despite its emphasis on the human–animal–environment relationship, the One Health approach can suffer from imbalanced representation and a narrow human‐centric focus. A systematic analysis of 100 unique One Health networks from 2018 revealed that approximately 31% engaged with only two sectors, with the environmental sector being the most underrepresented (Figure 1; Khan et al., 2018). Despite being critical to understanding and tackling public health threats, neglect of the environmental component of One Health is a recognised, long‐standing issue (Essack, 2018). Prevention and response strategies for dealing with pandemics, such as enhanced surveillance and vaccine development, are essential, but they do not address the underlying causes of these threats. Greater consideration should therefore be given to the anthropogenic influences on the environment that precede these public health crises.

Antimicrobial resistance mitigation is an enduring challenge but the role of the environment in increasing the risk of AMR is poorly understood and underrepresented in policies aimed at tackling the problem (Ogyu et al., 2020). Although the evolution of resistance occurs naturally over time, human activities (including release of pharmaceuticals from drug manufacturing processes, hospitals, as well as human and livestock waste; Azuma et al., 2019; Fouz et al., 2020; Wang et al., 2015) are increasing exposure to AMR bacteria and AMR genes. In turn, the risks of environmental AMR transmission are increased, and the AMR process is accelerated. The role of the environment as a potential reservoir for AMR is becoming increasingly recognised. However, environmental‐sector involvement in One Health networks is often lacking, and this impedes progress towards a true One Health approach. To this end, AMR studies are focusing on wastewater treatment plants to determine levels of AMR resistance expelled into these environments. Recent successful SARS‐CoV2 monitoring of sewage has determined the potential of environmental surveillance of COVID‐19 as an additional measure to infection monitoring in the population (Larsen & Wigginton, 2020).

Globally, the leading One Health approach involves collaboration between the World Health Organization (WHO), the Food and Agriculture Organization (FAO), and the World Organization for Animal Health (OIE). Although representation from the human and animal health sectors has been achieved, the environmental component of One Health appears neglected. Suggestions have been made to expand the collaboration to include the UN Environment Program to ensure representation from the environmental sector (de Garine‐Wichatitsky et al., 2020). The role of the environment in disease development and dissemination cannot be overlooked, and this is key to tackling public health threats such as AMR and COVID‐19. Moreover, with the contribution of communication to successful One Health collaborations (Errecaborde et al., 2019) in mind, we now consider public risk communication in response to global health threats.

## 4 PUBLIC RISK COMMUNICATION

In response to the COVID‐19 pandemic, public health authorities around the world have focused on the common goal of reducing virus transmission and disease mortality. However, health authorities have applied slightly different approaches in their efforts to protect public health and simultaneously cope with socioeconomic challenges. Most countries have implemented multiple measures including total lockdowns, travel bans, closure of non‐essential retail, social distancing, mandatory face covering, hand hygiene, and deep cleaning (European Centre for Disease Prevention & Control, 2021). Nevertheless, differences in the public risk communication of these measures have been observed. The purposes of the measures and the communication strategies have been to protect public health and enhance public understanding of viral transmission and prevention. As a result, countries have witnessed reductions in cases and decreases in the COVID‐19 basic reproduction (R_0_) number (Oraby et al., 2021). Smaller R_0_ numbers, typically indicating reduced infection transmissibility, have enabled periodic easing of some imposed restrictions. Equally, steady increases in the R0 number have required the reimposition of restrictions and communication of the changes (Han et al., 2020).

In January 2020, news emerging from Wuhan, China, of the novel virus with pneumonia‐like symptoms was met with concern and uncertainty. Early communications were that only those who had visited the Wuhan seafood market were likely to be at risk and that there was no evidence to suggest human‐to‐human transmission (Zhang et al., 2020). Contradicting theories regarding the origin of the virus and reporting delays from officials in China created distrust in the Chinese government. The reporting delays also meant that health workers and the public were unprepared to deal with the increasing severity of the novel coronavirus. As the virus spread globally, success and public acceptance of government response strategies varied from country to country. In the UK, for example, early perception of risk was low, and the government was slow to implement measures to minimise the spread of the virus. The downplaying of uncertainties (e.g. regarding virus doubling rate; Pearce, 2020), lack of transparency (e.g. initial non‐disclosure of expert adviser identities), and confused public health messaging further affected UK public trust in the ability of the government to control the pandemic. Early news coming from across the world in 2020 also led people to mistakenly believe that the virus affected mostly the vulnerable and the elderly (Paulik et al., 2020). Conflicting and unclear messages, including differing guidance on the use of face masks in the US (Nagler et al., 2020) and contradictory claims on the effectiveness of hydroxychloroquine across several countries (Belayneh, 2020; Nagler et al., 2020; Pulla, 2020), continued into later months of the pandemic. With the vaccine rollout in early 2021, there were reports of an increase in COVID‐19 infections among vaccinated people in Israel and the UK (Day, 2021). These spikes may have been due to non‐adherence to restrictions because individuals could have been unclear about the immediate immunity offered by vaccines.

### 4.1 Key features of effective public risk communication

Countries (such as New Zealand) that implemented the strictest restrictions on movement and social interaction saw some of the smallest number of COVID‐19 cases and associated deaths (Kung et al., 2021). Social distancing measures and early bans on international travel, extending to full border closures (as the pandemic progressed), were key to their success (Kung et al., 2021). New Zealand, generally having a reputation for good practice on the crisis management of the pandemic, provided clear and scientific risk‐infused communication, which contributed, initially, to suppress viral transmission (Jamieson, 2020). Positive and empathetic human‐focused messaging aided public understanding, created a sense of community, and promoted collective action (Hunt, 2021). What appears to be key in the approaches used by governments to enhance public understanding and acceptance of COVID‐19 control measures is the ability of governments to communicate risk promptly, effectively, and unambiguously. Effective communication involves openness and accessibility to risk information, developed through a message‐centred approach. It should involve a dynamic and interactive process of collaborative dialogue between governments, industry (including scientists and public health experts), and the public (Sellnow et al., 2009). Critical to the uptake of any message, though, is trust in a country's leadership (Paulik et al., 2020). Factors that support trust‐building include consistency and transparency of communication (Paulik et al., 2020), both of which were observed in Australia and New Zealand's risk communication approach and may have contributed to successful message adherence by the public. Transparent relationships between scientists, public health authorities, and the public need to be achieved to ensure effective communication that is based on well‐informed decisions. That said, there are uncertainties about the sustainability of consistent public health messaging, which is acutely important for public health threats like AMR. The 2020–2021 pandemic has demonstrated that ineffective communication, and its resulting poor public understanding, may be further fuelling AMR. For example, the efficacy of antimicrobial sprays depends on the contact time of the antimicrobial agent with the microbe(s) (West et al., 2018). However, ‘spray and wipe’ has been regarded as the required practice to disinfect surfaces, potentially leading to a rise in AMR to these agents. This suggests that clearer messaging is required to enhance public understanding and to prevent AMR‐associated outcomes. Success in the mitigation of AMR hinges on the ability of the public to understand its development and to implement appropriate behaviours that can reduce the risks of AMR.

The COVID‐19 pandemic response has revealed that risk communication that is timely, consistent, transparent, and from a trusted source is crucial to public engagement and acceptance. Lessons learned from communication plans and processes applied during the COVID‐19 pandemic should be used to refocus attention on AMR. The consistent message‐centred approach applied in COVID‐19 response should be considered when addressing the AMR challenge. Although consistency in messaging is important, repetitiveness and overexposure should be avoided to lessen public fatigue towards health risk messaging (Koh et al., 2020). The coronavirus pandemic has revealed that a delicate balance must be struck when communicating public health risks. However, this balance has, unfortunately, been tipped towards insufficient public health messaging about the implications of AMR and other global health threats. The pandemic has also tipped the balance against active response to these threats, which is considered in the next section of the article.

## 5 THE RISKS OF OVERSHADOW: HOW COVID‐19 RISKS DEPRIORITISING OTHER GLOBAL HEALTH THREATS

The pandemic has dominated global health research and disrupted responses to global threats that intersect with it, as limited available resources throw up the challenge of balancing immediate COVID‐19 response needs with those of other concurrently occurring crises, which has implications for the achievement of sustainable development goals for 2030 (Padma, 2020). Enduring health threats, such as AMR, are at risk of being deprioritised by the COVID‐19 pandemic. The redistribution of laboratory staff, equipment, and chemicals (Department of Health & Social Care, 2020) may interrupt ongoing AMR research and affect global monitoring systems. The pandemic appears to have loosened antimicrobial stewardship practices through increases in antibiotic prescriptions and pausing of hospital surveillance activities (Langford et al., 2021; Wellcome, 2020). Furthermore, constraints owing to COVID‐19 priorities could derail the evaluation phase of AMR national action plans developed between 2016 and 2017 (WHO, 2021g), potentially affecting future strategies. There is also need to draw attention to the pandemic‐induced delay in efforts aimed at reducing environmental antibiotic pollution from pharmaceutical manufacturers (Patnaik, 2020). Similarly, in mid‐2020, concern for AMR had fallen from 29% in the previous year to 9%, whereas pandemics and infectious diseases were regarded as the top emerging risks (AXA, 2020).

As noted earlier, the pandemic has derailed the achievement of the United Nations (UN) sustainable development goals (SDGs) for 2030, and threatens to reverse years of progress made towards achieving these (Safitri et al., 2021; United Nations, 2020). Disruptions to health services and health infrastructure brought on by COVID‐19 limit the approaches that can be adopted to realise the SDGs, many of which are related to the mitigation of AMR. For example, before the pandemic, the world was already off track to meet the 1.5–2°C temperature rise limit set by the Paris Agreement (World Meteorological Organization, 2021). Concerns over the ability to achieve this goal have been further amplified by the postponement of the UN climate change conference, and the temporary relaxation of environmental law enforcement by the US Environmental Protection Agency (EPA). As a result, industries may delay reporting greenhouse gas emissions while dealing with COVID‐19 (EPA, 2020). Maintaining progress towards achieving climate change goals is crucial to AMR, with increasing temperatures influencing the spread of resistance (Rodríguez‐Verdugo et al., 2020). Furthermore, there have been pandemic‐related disruptions to non‐COVID‐19 services in up to 70 countries (The Lancet Public Health, 2020), and this will negatively affect global health and well‐being in the coming years (Razai et al., 2021; United Nations, 2020). It is therefore crucial that measures adopted during the COVID‐19 pandemic have a strong degree of global policy alignment towards achieving the sustainable development goals.

### 5.1 Maintaining priorities by strengthening underlying systems: The Global Health Security Agenda

Pandemics and concurrently existing crises highlight the intertwined health, social, and economic challenges that must be managed in the midst of contracting economies and competing needs. Countries should use the opportunity presented by these crises to address the underlying systemic and structural issues that connect them. For example, Nigeria had some capacities to intensify its response to the COVID‐19 pandemic by utilising existing structures (including surveillance, logistics, and equipment) that had been established for their polio and Ebola epidemics (Ebenso & Otu, 2020). Strengthening underlying health, social care, and disaster management systems corresponds to the goals of the Global Health Security Agenda (GHSA), a framework that aims to help countries strengthen their public health systems to enhance their prevention, detection, and response capacities in addressing infectious disease threats (CDC, 2014). Focusing on AMR, the GHSA identifies AMR as a public health threat of concern, corroborated by the dedication of one of 11 GHSA action packages to AMR. Adopting GHSA and committing to the AMR action package will strengthen the ability of countries to devise comprehensive plans, underpinned by the One Health approach, to address antimicrobial resistance across all sectors (CDC, 2014). Alignment and integration of AMR into a pandemic preparedness agenda should also be considered as a way to focus on AMR and prevent its deprioritisation. The WHO has published updated guidance that integrates antimicrobial stewardship in the clinical management of the pandemic (WHO, 2021d). However, there are concerns over potential losses of distinct AMR activities and if the two areas can be linked (Wellcome, 2020). The agenda that best serves AMR requires more deliberation, and shorter‐term goals do not continue to deprioritise or undermine efforts to secure long‐term global health goals. Notionally, crisis preparedness and management should be approached holistically, and enable the application of integrated multisectoral approaches across several crises. The new international treaty for pandemic preparedness and response that is being proposed by leaders in government and global agencies should provide guidance and support for this (WHO, 2021f). This holistic approach will also ensure that solutions to one crisis do not negatively affect progress or exacerbate challenges in other areas. Effective multistakeholder collaboration between and within sectors will be key to achieving this. The article now turns to this dimension in more detail.

## 6 THE NEED FOR COLLABORATIVE GOVERNANCE AS ESSENTIAL AND NOT AS AN OPTIONAL EXTRA

**FIGURE 2:**
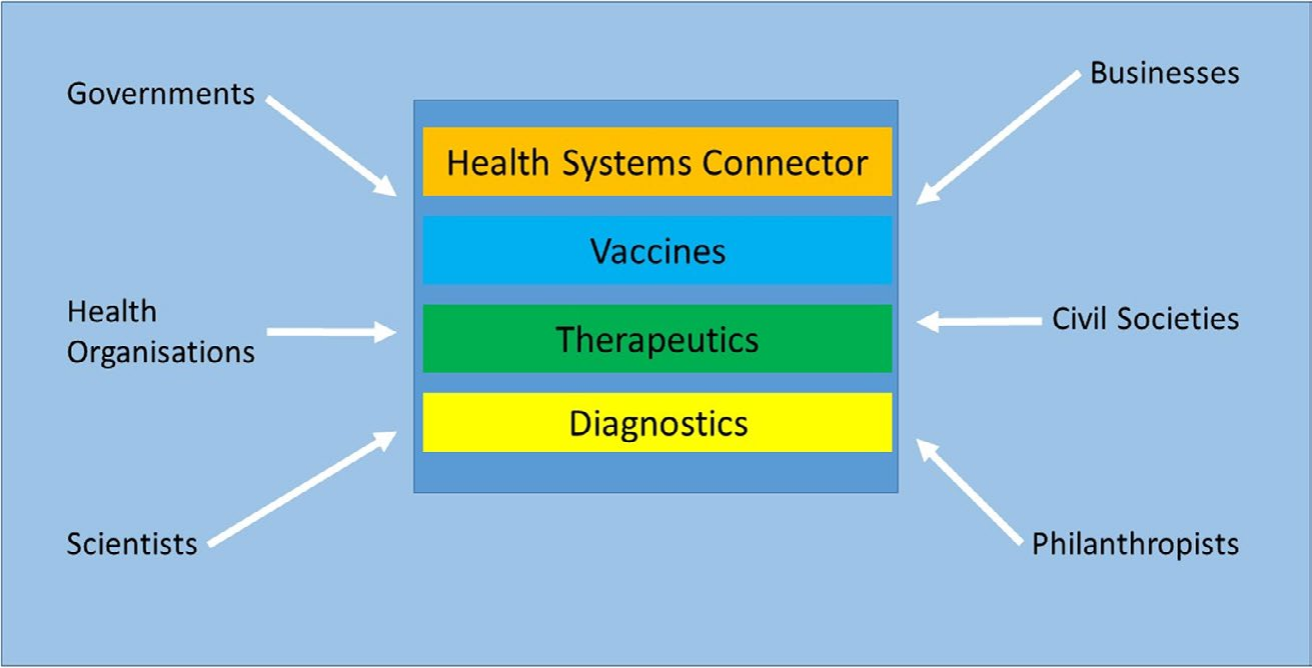
Access to COVID‐19 Tools (ACT) Accelerator Framework for collaboration

Collaborative governance is broadly defined as the processes associated with public policy decision‐making that involves engagement of stakeholders from public, civic, and private sectors to achieve a purpose (Emerson et al., 2012). The importance of collaborative governance at all levels (i.e. global, supranational, national, subnational, and local) has been emphasised by the COVID‐19 pandemic. The pandemic prompted concerted efforts to quell the acute phase of the disease spread (WHO, 2021h) and the scaling‐up of multiple interventions at global and supranational levels (Ansell & Torfing, 2018). Collaborative efforts adopted at different governmental levels have produced new systems that are specifically devoted to the pandemic. One such system is the Access to COVID‐19 Tools (ACT) Accelerator, a global framework for collaboration that involves partnerships between global health organisations and brings together governments, health organisations, researchers, academics, civil service, private stakeholders, and philanthropists (WHO, 2021b; see Figure 2). It was launched to strengthen health systems; facilitate rapid development of novel and effective COVID‐19 diagnostic devices, therapies, and vaccines; ensure production scale‐up of COVID‐19 tools; and facilitate equitable global access to these resources (WHO, 2021a, 2021h). Intergovernmental collaboration at this global level is made necessary by shared interests in developing tools to address the COVID‐19 pandemic.

Similar collaborative efforts in response to the COVID‐19 pandemic are observed at the supranational level. This includes the EU vaccine strategy adopted by the EU and its member states. Implementation of a single response at this supranational level is necessary because the EU single market allows the spread of the socioeconomic and health impact of the COVID‐19 pandemic across its member states (European Commission, 2021a). The EU vaccine strategy was developed, therefore, to secure timely and sufficient supplies of safe and effective vaccines, and ensure equitable access across its member states (European Commission, 2021a). This joint effort at the EU level enhances chances of success by increasing the capacities of EU member states to support vaccine research, allowing them to share risks and pool investments to achieve economies of scale (European Commission, 2021a). Collaborative efforts at international levels (e.g. the new virtual UK–India vaccines hub (GOV.UK, 2021b), national levels (e.g. public health campaigns, COVID‐19 Genomics UK (COG‐UK) consortium), and subnational levels (e.g. population surveillance programmes [GOV.UK, 2021a]), and planning for post‐pandemic economic recovery (Council, 2020) have also been critical to the COVID‐19 responses. These responses highlight the gains of concerted effort in situations that require coordinated activities, joint structures, and pooled resources (Müller & Van Esch, 2020). The responses also highlight the need for diverse expertise and experience, which allow the incorporation of viewpoints that generate practical solutions for effective resolution of this shared problem (Gardner & Matviak, 2020).

Barriers to collaboration need to be identified and overcome in order for collaborations to have meaningful outcomes. As corroborated by the COVID‐19 response, emphasis on shared objectives, establishing and maintaining good communication between partners, and promoting equitable and timely resource sharing should lead all collaborative endeavours (Commission, 2021). In addition, it is important that individual bodies recognise their limited capacity to independently address global health threats, because this will reduce subscription to self‐interested measures (Bump et al., 2021; European Commission, 2021b).

The experience of the pandemic indicates, strongly, that national self‐interest played a central role in hampering initial COVID‐19 responses and widened inequalities between the Global North and the Global South in terms of vaccine coverage and distribution. International cooperation has been impeded by political tensions concerning the origins of the novel coronavirus (Relman, 2020) and potential cover‐up of the scale of disease fatality (Cukierman, 2020). Border closures, travel bans, and export restrictions imposed by certain countries limited import capacity of countries (as they searched for scarce resources; OECD, 2020) and hampered national and global efforts to contain the pandemic (Braw, 2020). Consequently, in early 2021, some countries secured enough vaccines to vaccinate their population many times over, whereas other countries were successful in securing vaccines for only a small fraction of their population (Mwai, 2021). These COVID‐19 responses highlight the need for the moral handling of public health threats; otherwise, inequalities will continue to widen.

### 6.1 Strengthening the role of global institutions for AMR

Global institutions, such as the WHO, play key roles during global health crises owing to the borderless health and socioeconomic impacts of these crises (Pegram & Kreienkamp, 2020). The role of the WHO has been critical during the COVID‐19 pandemic. However, challenges faced by the WHO—including inadequate financing to deploy support and resources, a cumbersome global pandemic alert system, and ineffective member‐state engagement with the WHO—meant that the WHO was underpowered to effectively carry out the job expected of it (Independent Panel for Pandemic Preparedness & Response, 2021). The WHO claims that it is drawing on lessons (from COVID‐19 and other prior health emergencies) to make improvements for the future and empower countries and global agencies to successfully manage public health threats (Independent Panel for Pandemic Preparedness & Response, 2021). The WHO need to be empowered to provide global leadership to manage global health threats, including AMR. Moreover, the pandemic offers a glimpse of the far‐reaching disruptions to global health, food security, and global economy that AMR can create (Strathdee et al., 2020; WHO, 2020). It is therefore necessary to apply some lessons learned from the COVID‐19 pandemic to avoid the devastating consequences of AMR. One lesson is the need for transdisciplinary collaboration to tackle the multidimensional challenges associated with AMR. As with the COVID‐19 pandemic, focus should be on strengthening health systems, creating strong public health campaigns that demonstrate the contribution of human behaviours to the development of AMR, and establishing resilient AMR surveillance programmes that estimate the AMR burden (WHO, 2020). Collaborations should also be established to identify emerging resistance and spread, promote antimicrobial stewardship, develop novel antimicrobials effective against resistant strains and vaccines to protect against infection, and implement measures to promote equitable global access to antimicrobials and vaccines (Jinks et al., 2016; Strathdee et al., 2020; WHO, 2020).

## 7 SOCIAL‐SCIENCE RESEARCH IS FUNDAMENTAL

Social‐science research is fundamental to addressing health threats and global security from a One Health perspective (Ruckert et al., 2020). COVID‐19 has illuminated the fact that threats such as AMR cannot be addressed unless understanding of societal, organisational, community, and individual behaviours is at the forefront of research studies and public policy responses (Bavel et al., 2020; Shah, 2020; Vedadhir et al., 2020). Pre‐COVID‐19 research has highlighted that the integration of social science into infectious disease epidemic preparedness and response, as well as into AMR research, has been peripheral and fragmentary (Bardosh et al., 2020; Frid‐Nielsen et al., 2019). AMR is yet to develop an identity in social‐science journals, as publications on AMR tend to be in interdisciplinary journals, which lean more towards the natural sciences. The global health challenge of COVID‐19 has reinforced the need for a broader, multidisciplinary approach to global health security in which social science is an integrated component rather than limited to a siloed means to promote community adherence to policies. It has been argued, convincingly, that global health security strategies should be underpinned by analysis of threat perception, social psychology, and behavioural analysis (Bavel et al., 2020). These perspectives can also serve fundamental uses in terms of informing science communication strategies (including understanding roots and implications of ‘fake news’/conspiracy theories) and with regards to evaluating the ethical considerations of health security interventions in governance systems. A challenge for social scientists is to respond to new breakthroughs and keep abreast of new/emerging understandings and findings. In many senses, this calls for the institutionalisation of social‐science leadership and expertise in government, particularly health agencies who can fulfil the role of both researcher and knowledge broker/organiser (Connolly et al., 2020; Meyer, 2010). In this respect, social scientists are key for horizon scanning and implementing knowledge resilience in an attempt to draw together the most appropriate data at the right time and communicate the data in the right format. Yet, this should form part of the internal workings of government agencies.

Furthermore, the pandemic has revealed that social‐science‐based analyses are required at multiple levels. This includes how individuals will behave in response to government‐led communications about, for instance, mask‐wearing and/or social distancing (Barrios et al., 2020); whether the levels of trust in government will have implications for the adherence of control measures (Balog‐Way & McComas, 2020; Connolly, 2020b); understanding the unintended health outcomes of policy responses to the pandemic (including for mental health and the implications for inequalities Bambra et al., 2020; Kumar & Nayar, 2020); as well as how, and what forms, of state governance need to adapt during (and after) such a prolonged crisis (Connolly & van der Zwet, 2021; Yang, 2020). These matters of ‘governance’ are also about the relationship between the state and its citizens. This, of course, is fundamental to the work of political scientists.

### 7.1 Social science: Bringing lessons together for the future of AMR global governance

Social‐science perspectives need to be at the forefront of lesson learning for the future. This includes refracting political science perspectives into the processes to understand the lessons that need to be learned. For example, political scientists are crucial to examining crisis management capacities in government and public attitudes to crisis measures (such as social distancing) and framing dynamics around blame‐gaming over the causes of pandemics. Governance frailties undermine global collaborative working for dealing with the ‘causes of the causes’ of such outbreaks (e.g. poor sanitation and living conditions and poverty). Without insights from scholarship on psychology, public administration, social policy, economics, politics, sociology, anthropology (to name just a few), the ability to address the causes of the causes of health threats are likely to be limited (Marmot, 2018). Furthermore, evaluation methodologies (including piloting approaches) are needed to determine how, and in what ways, individual geographically focused One Health‐based interventions can be scaled up (Connolly, 2020, 2020a). The issue of scale is important because in order to scale interventions there needs to be situational and contextual analyses of the barriers to and enablers of drawing lessons from one territory or governance level (e.g. macro, meso, or micro) and applying them in another. In this respect, the social sciences are fundamental to understanding how the science of AMR fits (or otherwise) within the policy and regulatory cultures and norms in different political environments.

The ‘value added’ of the social sciences is not just about how scientific findings can be better disseminated across policy systems; rather, social science research can direct where scientific research can, or should, take place in the first place. This might be identifying where and how to co‐create interventions with civil society stakeholders, which requires trust building with communities. AMR research often takes place in areas where there might be cultural sensitivities and trust barriers between communities and researchers (Doron & Broom, 2019). Social researchers need to be conscious of their identities and adapt their behaviours as a result. This reinforces the point that a great deal of AMR research is about understanding how to navigate complex relationships in sensitive, and sometimes risky, policy and cultural spaces (Marks & Abdelhalim, 2018). Achieving buy‐in from those parties who ultimately need to change their behaviours, including industry, policymakers, and communities is crucial, and, in fact, is a matter for social scientists. The evaluation research literature has highlighted that failing to be co‐creative with stakeholders will mean that research will fall short of achieving an impact in that dissemination risks being seen as an afterthought rather than being ‘utilisation focused’ (Michael Quinn Patton, 2008; Sager et al., 2020). Impact needs to be embedded from the policy, project, or policy design phase. It is far more customary within contemporary research applications (Research Funders, for instance) for impact statements to encourage this type of approach; yet, this serves to further enhance the importance of the social sciences—particularly in undertaking social and political network mapping of the institutions and access points for forming collaborations to influence policymakers. On this basis, there are opportunities for AMR research to capitalise on the important acknowledgement of the critical importance of the social sciences in pandemic research (Bavel et al., 2020). This is not to say, however, that the social sciences have not made important synergies within AMR research. A recent systemic review has documented the fact that social‐science‐based AMR research can be seen in several areas, ranging from surveillance and risk assessment of AMR to the communication dimensions of the most appropriate use of antimicrobials in primary care and clinical settings (Lu et al., 2020). A conclusion that can be drawn from this research is that more One Health studies should be frontloaded by social‐science research.

## 8 CONCLUSIONS: A NEW PARADIGM FOR AMR‐FOCUSED GLOBAL HEALTH SECURITY

The COVID‐19 pandemic has caused global disruptions at unimaginable scales and exposed the deficiencies of existing structures. As this is unlikely to be the last global health threat that the world will face, there are lessons to be learned that will improve preparedness and response to other global crises. Global COVID‐19 pandemic responses have highlighted the need for consistent, transparent, and clear messaging from trusted sources to enhance public engagement, acceptance, and cooperation. Like COVID‐19, effective communication is critical to achieving success in AMR mitigation, because it will enhance public understanding of factors that exacerbate AMR and behaviours that mitigate it. The pandemic also revealed shortcomings in the global health space, which has been evident in the deprioritisation of concurrently existing global crises, such as AMR. Understanding the underlying systemic and structural issues that precede and connect crises is crucial to the effective mitigation of future public health risks.

Given the scale of AMR, no state can manage it on their own. There is the need for collaboration across all levels of government, with the understanding that ‘no one is safe, unless everyone is safe’ (WHO, 2021c). With this understanding, governments should unsubscribe from isolationism, reaffirm their commitment to multilateral cooperation, and provide support to global health organisations. The One Health approach should be identified as the primary foundation for public health policy. Going forward, implementation of the One Health approach should encourage greater representation from the environmental and social sciences. This will facilitate greater collaborative capacities and enhance understanding of the entire landscape of multisectorial contributions to public health. Social‐science perspectives will be critical in bringing the above lessons together for the future of global health. Understanding the barriers to and enablers of effective communication and trust between scientists, policymakers, and the public is essential to achieving buy‐in from stakeholders and communities. Importantly, navigation of the complex governance relationships in different political cultures and environments to tackling the ‘causes of the causes’ of public health threats, such as AMR, needs to be prioritised. Social science research can aid the understanding of these relationships, facilitate collaborative efforts, and should therefore be at the forefront of global health security.

The COVID‐19 pandemic, and other prior health emergencies (e.g. severe acute respiratory syndrome [SARS], Middle East respiratory syndrome [MERS], and Ebola), have provided opportunities for leaders (at all levels) to institute baseline structures to prepare and respond to AMR and other global threats. Leaders need to direct resources towards strengthening public and environmental health, including environmental monitoring systems, social care, and disaster management systems to ensure that, at their core, health systems are collaborative, strong, and resilient. This, as well as the above lessons learned from the COVID‐19 pandemic, could be implemented in the context of the GHSA. Adoption of this framework, therefore, will enable countries to scale up their prevention, detection, and response capacities to public health threats and allow countries to shift from disease‐focused approaches (where trade‐offs might be required during competing crises), to a systems‐focused approach (where existing structures can support concurrently existing threats). With specific focus on AMR, the implementation of the action items in the Antimicrobial Resistance action package of the GHSA would help address antimicrobial resistance in human and animal sectors. Integration of AMR activities in other agendas, such as the proposed international treaty for pandemic preparedness agenda, will also be beneficial. Bearing in mind that this proposed agenda would be underpinned by the International Health Regulations, it is expected that activities in the GHSA action packages will be in line with the proposed treaty for pandemic preparedness, providing opportunities for the exploration of synergies in both agendas. The disproportionate effects of pandemics and other global health threats require leaders to build equitable and inclusive economies and strong systems that are resilient to global crises. Therefore, the key messages to take from this paper are:

- The One Health approach suffers from a narrow human‐centric view, with the environmental sector being the least represented in AMR policy.
- Public risk communication has differed across countries. Messages that are timely, consistent, transparent, and from a trusted source crucial to public understanding and acceptance of health measures.
- Agendas need to be aligned and underlying health systems need to be strengthened to avoid deprioritisation of coexisting global health threats such as AMR.
- Collaborative forms of governance, and strengthened roles for global institutions, are essential in public health crisis management.
- Social science is fundamental to health security policymaking and governance processes.

## References

[gpol13049-bib-0001] Ansell, C. & Torfing, J. (Eds.) (2018) How does collaborative governance scale?. Bristol, UK: Policy Press. 10.1332/policypress/9781447340553.001.0001

[gpol13049-bib-0002] AXA . (2020) AXA future risks report contents. Available at: https://www.axa.com/en/press/publications/future‐risks‐report‐2020 [Accessed 22 March 2021]

[gpol13049-bib-0003] Azuma, T. , Otomo, K. , Kunitou, M. , Shimizu, M. , Hosomaru, K. , Mikata, S. et al. (2019) Environmental fate of pharmaceutical compounds and antimicrobial‐resistant bacteria in hospital effluents, and contributions to pollutant loads in the surface waters in Japan. Science of the Total Environment, 657, 476–484. 10.1016/j.scitotenv.2018.11.433 30550911

[gpol13049-bib-0004] Balog‐Way, D.H.P. & McComas, K.A. (2020) ‘COVID‐19: Reflections on trust, tradeoffs, and preparedness. Journal of Risk Research, 23(7–8), 838–848. 10.1080/13669877.2020.1758192

[gpol13049-bib-0005] Bambra, C. , Riordan, R. , Ford, J. & Matthews, F. (2020) The COVID‐19 pandemic and health inequalities. Journal of Epidemiology and Community Health, 74, 964–968. 10.1136/jech-2020-214401 32535550 PMC7298201

[gpol13049-bib-0006] Bardosh, K.L. , de Vries, D.H. , Abramowitz, S. , Thorlie, A. , Cremers, L. , Kinsman, J. et al. (2020) Integrating the social sciences in epidemic preparedness and response: A strategic framework to strengthen capacities and improve Global Health security. Globalization and Health, 16(1), 120. 10.1186/s12992-020-00652-6 33380341 PMC7772799

[gpol13049-bib-0007] Barrios, J. , Benmelech, E. , Hochberg, Y.V. , Sapienza, P. & Luigi . (2020) Civic Capital and Social Distancing during the Covid‐19 Pandemic. Cambridge, MA: National Bureau of Economic Research.

[gpol13049-bib-0008] Barton Behravesh, C. (2019) Introduction. One Health: over a decade of progress on the road to sustainability. Revue Scientifique Et Technique (International Office of Epizootics), 38(1), 21–50. 10.20506/rst.38.1.2939 31564742

[gpol13049-bib-0009] Bavel, J.J.V. , Skitka, L.J. , Smith, S.S. , Sunstein, C.R. , Tabri, N. , Tucker, J.A. et al. (2020) Using social and behavioural science to support COVID‐19 pandemic response. Nature Human Behaviour, 4(5), 460–471. 10.1038/s41562-020-0884-z 32355299

[gpol13049-bib-0010] Belayneh, A. (2020) Off‐label use of chloroquine and Hydroxychloroquine for COVID‐19 treatment in Africa against WHO Recommendation. Research and Reports in Tropical Medicine, 11, 61–72. 10.2147/rrtm.s269936 32982538 PMC7505701

[gpol13049-bib-0011] Boin, A. , McConnell, A. & ‘ t Hart, P. (2021) Governing the pandemic. Cham: Springer International Publishing. 10.1007/978-3-030-72680-5

[gpol13049-bib-0012] Braw, E. (2020) Europe’s coronavirus response: selfish member states and active institutions, RUSI. Available at: https://rusi.org/commentary/europe‐coronavirus‐response‐selfish‐member‐states‐and‐active‐institutions [Accessed 3 March 2021]

[gpol13049-bib-0013] Bump, J.B. , Friberg, P. & Harper, D.R. (2021) ‘International collaboration and covid‐19: what are we doing and where are we going? BMJ, n180. 10.1136/bmj.n180 33509953 PMC7842258

[gpol13049-bib-0014] Cairney, P. & Wellstead, A. (2020) COVID‐19: effective policymaking depends on trust in experts, politicians, and the public. Policy Design and Practice, 9, 1–14. 10.1080/25741292.2020.1837466

[gpol13049-bib-0015] CDC . (2014) Global health security agenda: action packages. Available at: https://www.cdc.gov/globalhealth/healthprotection/ghs/pdf/ghsa‐action‐packages_24‐september‐2014.pdf [Accessed 18 May 2021]

[gpol13049-bib-0016] Clancy, C.J. , Buehrle, D.J. & Nguyen, M.H. (2020) PRO: The COVID‐19 pandemic will result in increased antimicrobial resistance rates. JAC‐Antimicrobial Resistance, 2(3), 1–3. 10.1093/jacamr/dlaa049 PMC7454644 34192248

[gpol13049-bib-0017] Collignon, P. & Beggs, J.J. (2020) CON: COVID‐19 will not result in increased antimicrobial resistance prevalence. JAC‐Antimicrobial Resistance, 2(3), 1–3. 10.1093/jacamr/dlaa051 PMC7454599 34192249

[gpol13049-bib-0018] Commission, C.Q. (2021) How providers are working together across systems in response to COVID‐19. Available at: https://www.cqc.org.uk/publications/major‐reports/how‐providers‐are‐working‐together‐across‐systems‐response‐covid‐19 [Accessed 18 February 2021]

[gpol13049-bib-0019] Connolly, J. (2016) The politics and crisis management of animal health security. Oxon: Routledge. 10.4324/9781315554440

[gpol13049-bib-0020] Connolly, J. (2020) Public attitudes and the management of the COVID‐19 crisis: the importance of personal responsibility, British Politics and Policy at LSE. Available at: https://blogs.lse.ac.uk/politicsandpolicy/personal‐responsibility‐covid‐19/ [Accessed 19 March 2021]

[gpol13049-bib-0021] Connolly, J. (2020a) Global crisis leadership for disease‐induced threats: One Health and urbanisation. Global Policy, 11(3), 283–292. 10.1111/1758-5899.12806 PMC7228258 32427190

[gpol13049-bib-0022] Connolly, J. , Reid, G. , Knoll, M. , Halliday, W. & Windsor, S. (2020) The sustainability of knowledge brokerage of the mental health improvement outcomes framework in Scotland: a follow‐up analysis. Evidence & Policy: A Journal of Research, Debate and Practice, 16(1), 177–195. 10.1332/174426418X15193815997735

[gpol13049-bib-0023] Connolly, J. & van der Zwet, A. (Eds.) (2021) Public value management, governance and reform in Britain. Cham: Springer International Publishing (International Series on Public Policy). 10.1007/978-3-030-55586-3

[gpol13049-bib-0024] Council, G.C. (2020) Post‐pandemic economic recovery plan for Glasgow to be developed. Available at: https://www.glasgow.gov.uk/article/25869/Post‐Pandemic‐Economic‐Recovery‐Plan‐for‐Glasgow‐to‐be‐developed [Accessed 18 February 2021]

[gpol13049-bib-0025] Cukierman, A. (2020) Why is COVID‐19 incidence in authoritarian China so much lower than in the democratic US: Effectiveness of collective action or Chinese cover‐up? | VOX, CEPR Policy Portal. VOX EU. Available at: https://voxeu.org/content/why‐covid‐19‐incidence‐authoritarian‐china‐so‐much‐lower‐democratic‐us‐effectiveness‐collective‐action‐or‐chinese‐cover [Accessed 18 March 2021]

[gpol13049-bib-0026] Day, M. (2021) Covid‐19: Stronger warnings are needed to curb socialising after vaccination, say doctors. BMJ, 372, 783. 10.1136/bmj.n783 33741565

[gpol13049-bib-0027] Department of Health and Social Care . (2020) Guidance for organisations seeking to support the COVID 19 testing programme department of health and social care. Available at: https://www.gov.uk/government/publications/coronavirus‐covid‐19‐scaling‐up [Accessed 22 March 2021]

[gpol13049-bib-0028] Devlin, M. (2020) Antimicrobial resistance: the next pandemic?, Microbiology Society. Available at: https://microbiologysociety.org/blog/antimicrobial‐resistance‐the‐next‐pandemic.html [Accessed 20 May 2021]

[gpol13049-bib-0029] Dhama, K. , Patel, S.K. , Sharun, K. , Pathak, M. , Tiwari, R. , Yatoo, M.I. et al. (2020) SARS‐CoV‐2 jumping the species barrier: Zoonotic lessons from SARS, MERS and recent advances to combat this pandemic virus. Travel Medicine and Infectious Disease, 37, 101830. 10.1016/j.tmaid.2020.101830 32755673 PMC7396141

[gpol13049-bib-0030] Doron, A. & Broom, A. (2019) The spectre of superbugs: waste, structural violence and antimicrobial resistance in India. Worldwide Waste: Journal of Interdisciplinary Studies, 2(1), 1–10. 10.5334/wwwj.20

[gpol13049-bib-0031] Ebenso, B. & Otu, A. (2020) Can Nigeria contain the COVID‐19 outbreak using lessons from recent epidemics? 10.1016/S0140-6736(20)30411-6 PMC7104043 32171055

[gpol13049-bib-0032] Emerson, K. , Nabatchi, T. & Balogh, S. (2012) An integrative framework for collaborative governance. Journal of Public Administration Research and Theory, 22(1), 1–29. 10.1093/jopart/mur011

[gpol13049-bib-0033] EPA . (2020) EPA announces enforcement discretion policy for COVID‐19 pandemic. Available at: https://www.epa.gov/newsreleases/epa‐announces‐enforcement‐discretion‐policy‐covid‐19‐pandemic [Accessed 22 March 2021]

[gpol13049-bib-0034] Errecaborde, K.M. , Macy, K.W. , Pekol, A. , Perez, S. , O’Brien, M.K. , Allen, I. et al. (2019) Factors that enable effective One Health collaborations ‐ A scoping review of the literature. PLoS One, 14(12), e0224660. 10.1371/journal.pone.0224660 31800579 PMC6892547

[gpol13049-bib-0035] Essack, S.Y. (2018) Environment: the neglected component of the One Health triad. The Lancet Planetary Health, 2(6), e238–e239. 10.1016/S2542-5196(18)30124-4 29880152 PMC7129022

[gpol13049-bib-0036] European Centre for Disease Prevention and Control . (2021) Data on country response measures to COVID‐19. Available at: https://www.ecdc.europa.eu/en/publications‐data/download‐data‐response‐measures‐covid‐19 [Accessed 09 December 2021]

[gpol13049-bib-0037] European Commission . (2021a) Communication from the Commission. EU Strategy for COVID‐19 vaccines. Available at: https://eur‐lex.europa.eu/legal‐content/EN/TXT/?uri=CELEX%3A52020DC0245 [Accessed 18 February 2021]

[gpol13049-bib-0038] European Commission . (2021b) The European single market | Internal market, industry, entrepreneurship and SMEs. Available at: https://ec.europa.eu/growth/single‐market_en [Accessed 18 February 2021]

[gpol13049-bib-0040] Fouz, N. , Pangesti, K.N.A. , Yasir, M. , Al‐Malki, A.L. , Azhar, E.I. , Hill‐Cawthorne, G.A. et al. (2020) The contribution of wastewater to the transmission of antimicrobial resistance in the environment: implications of mass gathering settings. Tropical Medicine and Infectious Disease, 5(1), 33. 10.3390/tropicalmed5010033 PMC7157536 32106595

[gpol13049-bib-0041] Frid‐Nielsen, S.S. , Rubin, O. & Baekkeskov, E. (2019) The state of social science research on antimicrobial resistance. Social Science and Medicine. Elsevier Ltd, 242, 112596. 10.1016/j.socscimed.2019.112596 31654893

[gpol13049-bib-0042] Gardner, H. & Matviak, I. (2020) 7 Strategies for promoting collaboration in a crisis, Harvard Business Review. Available at: https://hbr.org/2020/07/7‐strategies‐for‐promoting‐collaboration‐in‐a‐crisis [Accessed 3 March 2021]

[gpol13049-bib-0043] de Garine‐Wichatitsky, M. , Binot, A. , Morand, S. , Kock, R. , Roger, F. , Wilcox, B.A. et al. (2020) Will the COVID‐19 crisis trigger a One Health coming‐of‐age? The Lancet Planetary Health, 4(9), e377–e378. 10.1016/S2542-5196(20)30179-0 32918881 PMC7480977

[gpol13049-bib-0045] GOV.UK . (2021a) COVID‐19 surveillance . Available at: https://www.gov.uk/government/publications/covid‐19‐surveillance/covid‐19‐surveillance [Accessed: 18 February 2021]

[gpol13049-bib-0046] GOV.UK . (2021b) UK and India to accelerate collaboration on vaccines to prevent future pandemics – GOV.UK. Available at: https://www.gov.uk/government/news/uk‐and‐india‐to‐accelerate‐collaboration‐on‐vaccines‐to‐prevent‐future‐pandemics [Accessed 18 February 2021]

[gpol13049-bib-0047] Han, E. , Tan, M.M.J. , Turk, E. , Sridhar, D. , Leung, G.M. , Shibuya, K. et al. (2020) Lessons learnt from easing COVID‐19 restrictions: an analysis of countries and regions in Asia Pacific and Europe. The Lancet, 396(10261), 1525–1534. 10.1016/S0140-6736(20)32007-9 PMC7515628 32979936

[gpol13049-bib-0048] Hitziger, M. , Esposito, R. , Canali, M. , Aragrande, M. , Häsler, B. & Rüegg, S.R. (2018) Knowledge integration in One Health policy formulation, implementation and evaluation. Bulletin of the World Health Organization, 96(3), 211–218. 10.2471/BLT.17.202705 29531420 PMC5840631

[gpol13049-bib-0049] Hsu, J. (2020) How covid‐19 is accelerating the threat of antimicrobial resistance. BMJ, 369, 1–2. 10.1136/bmj.m1983 32423901

[gpol13049-bib-0050] Hunt, E. (2021) Words matter: how New Zealand’s clear messaging helped beat Covid | New Zealand. The Guardian. Available at: https://www.theguardian.com/world/2021/feb/26/words‐matter‐how‐new‐zealands‐clear‐messaging‐helped‐beat‐covid [Accessed 24 March 2021]

[gpol13049-bib-0051] Independent Panel for Pandemic Preparedness and Response . (2021) Second report on progress. 10.1016/S0140-6736(21)01095-3 PMC9751704 33991477

[gpol13049-bib-0052] Jamieson, T. (2020) “Go hard, go early”: preliminary lessons from New Zealand’s response to COVID‐19. The American Review of Public Administration, 50(6–7), 598–605. 10.1177/0275074020941721

[gpol13049-bib-0053] Jinks, T. , Lee, N. , Sharland, M. , Rex, J. , Gertler, N. , Diver, M. et al. (2016) ‘A time for action: Antimicrobial resistance needs global response. Bulletin of the World Health Organization, 558–558A. 10.2471/BLT.16.181743 27516629 PMC4969997

[gpol13049-bib-0054] Kanankege, K.S.T. , Phelps, N.B.D. , Vesterinen, H.M. , Errecaborde, K.M. , Alvarez, J. , Bender, J.B. et al. (2020) Lessons Learned from the stakeholder engagement in research: application of spatial analytical tools in One Health problems. Frontiers in Veterinary Science, 7, 254. 10.3389/fvets.2020.00254 32478109 PMC7237577

[gpol13049-bib-0055] Khan, M.S. , Rothman‐Ostrow, P. , Spencer, J. , Hasan, N. , Sabirovic, M. , Rahman‐Shepherd, A. et al. (2018) The growth and strategic functioning of One Health networks: a systematic analysis. The Lancet Planetary Health, 2(6), e264–e273. 10.1016/S2542-5196(18)30084-6 29880158

[gpol13049-bib-0056] Kim, J. , Coble, D.J. , Salyards, G.W. , Bower, J.K. , Rinaldi, J.W. , Plauche, G.W. et al. (2017) Antimicrobial use for and resistance of zoonotic bacteria recovered from nonhuman primates, Comparative Medicine, 67(1), pp. 79–86. 28222842 PMC5310628

[gpol13049-bib-0057] Koh, P.K.K. , Chan, L.L. & Tan, E.K. Messaging fatigue and desensitisation to information during pandemic. Archives of Medical Research, 51(7), 716–717. 10.1016/j.arcmed.2020.06.014 PMC7377807 32713728

[gpol13049-bib-0058] Kumar, A. & Nayar, K.R. (2020) ‘COVID 19 and its mental health consequences. Journal of Mental Health, 30(1), 1–2. 10.1080/09638237.2020.1757052 32339041

[gpol13049-bib-0059] Kung, S. , Doppen, M. , Black, M. , Hills, T. & Kearns, N. (2021) Reduced mortality in New Zealand during the COVID‐19 pandemic. The Lancet, 397(10268), 25. 10.1016/S0140-6736(20)32647-7 PMC7833946 33333005

[gpol13049-bib-0060] Langford, B.J. , So, M. , Raybardhan, S. , Leung, V. , Soucy, J.‐P. , Westwood, D. et al. (2021) Antibiotic prescribing in patients with COVID‐19: rapid review and meta‐analysis. Clinical Microbiology and Infection, 27(4), 520–531. 10.1016/j.cmi.2020.12.018 33418017 PMC7785281

[gpol13049-bib-0061] Larsen, D.A. & Wigginton, K.R. (2020) Tracking COVID‐19 with wastewater. Nature Biotechnology, 38(10), 1151–1153. 10.1038/s41587-020-0690-1 PMC7505213 32958959

[gpol13049-bib-0062] Lu, J. , Sheldenkar, A. & Lwin, M.O. (2020) A decade of antimicrobial resistance research in social science fields: a scientometric review. Antimicrobial Resistance and Infection Control, 178, 178–191. 10.1186/s13756-020-00834-2 PMC7643349 33148344

[gpol13049-bib-0064] Marks, M. & Abdelhalim, J. (2018) Introduction: identity, jeopardy and moral dilemmas in conducting research in “risky” environments. Contemporary Social Science, 13(3–4), 305–322. 10.1080/21582041.2017.1388463

[gpol13049-bib-0065] Marmot, M. (2018) Inclusion health: addressing the causes of the causes. The Lancet, 391, 186–188. 10.1016/S0140-6736(17)32137-2 29137870

[gpol13049-bib-0066] Mazey, S. & Richardson, J. (2020) Lesson‐Drawing from New Zealand and Covid‐19: The Need for Anticipatory Policy Making. The Political Quarterly, 91(3), 561–570. 10.1111/1467-923X.12893 PMC7436465 32836413

[gpol13049-bib-0067] McConnell, A. (2011) Success? Failure? Something in‐between? A framework for evaluating crisis management. Policy and Society, 30(2), 63–76. 10.1016/j.polsoc.2011.03.002

[gpol13049-bib-0068] Meyer, M. (2010) The rise of the knowledge broker. Science Communication, 32(1), 118–127. 10.1177/1075547009359797

[gpol13049-bib-0069] Michael Quinn Patton . (2008) Utilization‐focused evaluation. New York: SAGE Publications Inc. Available at: https://us.sagepub.com/en‐us/nam/utilization‐focused‐evaluation/book229324 (Accessed 21 March 2021)

[gpol13049-bib-0070] Müller, H. & Van Esch, F.A.W.J. (2020) Collaborative leadership in EMU governance: a matter of cognitive proximity. West European Politics, 43(5), 1117–1140. 10.1080/01402382.2019.1678950

[gpol13049-bib-0071] Mwai, P. (2021) Covid‐19 Africa: Vaccine rollout is gathering pace – BBC News, 2021. Available at: https://www.bbc.co.uk/news/56100076 [Accessed 3 March 2021]

[gpol13049-bib-0072] Nagler, R.H. , Vogel, R.I. , Gollust, S.E. , Rothman, A.J. , Fowler, E.F. & Yzer, M.C. (2020) Public perceptions of conflicting information surrounding COVID‐19: Results from a nationally representative survey of US adults. PLoS One, 15(10), e0240776. 10.1371/journal.pone.0240776 33085719 PMC7577476

[gpol13049-bib-0073] O’Neill, J. (2016) Tackling drug‐resistant infections globally: Final report and recommendations. Available at: https://www.biomerieuxconnection.com/wp‐content/uploads/2018/04/Tackling‐Drug‐Resistant‐Infections‐Globally_‐Final‐Report‐and‐Recommendations.pdf [Accessed 19 March 2021]

[gpol13049-bib-0074] OECD (2020) Trade interdependencies in Covid‐19 goods. Available at: https://www.oecd.org/coronavirus/policy‐responses/trade‐interdependencies‐in‐covid‐19‐goods‐79aaa1d6/ [Accessed 3 March 2021]

[gpol13049-bib-0075] Ogyu, A. , Chan, O. , Littmann, J. , Pang, H.H. , Lining, X. , Liu, P. et al. (2020) National action to combat AMR: A One‐Health approach to assess policy priorities in action plans. BMJ Global Health, 5(7), 2427. 10.1136/bmjgh-2020-002427 PMC7359186 32665430

[gpol13049-bib-0076] One Health Commission (2021) Why One Health? Available at: https://www.onehealthcommission.org/en/why_one_health/ [Accessed 19 March 2021]

[gpol13049-bib-0077] One Health Global Network . (2015) What is One Health? Available at: http://www.onehealthglobal.net/what‐is‐one‐health/ [Accessed 19 March 2021]

[gpol13049-bib-0078] Oraby, T. , Tyshenko, M.G. , Maldonado, J.C. , Vatcheva, K. , Elsaadany, S. , Alali, W.Q. et al. (2021) Modeling the effect of lockdown timing as a COVID‐19 control measure in countries with differing social contacts. Scientific Reports, 11(1), 1–13. 10.1038/s41598-021-82873-2 33558571 PMC7870675

[gpol13049-bib-0079] Overgaauw, P.A.M. , Vinke, C.M. , van Hagen, M.A.E. & Lipman, L.J.A. (2020) A One Health perspective on the human‐companion animal relationship with emphasis on zoonotic aspects. International Journal of Environmental Research and Public Health, 17(11), 3789. 10.3390/ijerph17113789 PMC7312520 32471058

[gpol13049-bib-0080] Padma, T. (2020) When natural disasters cross the path of COVID‐19. Eos, 101. 10.1029/2020eo146481

[gpol13049-bib-0081] Patnaik, A. (2020) You thought COVID was a threat? Antimicrobial resistance is a close second. Available at: https://www.outlookindia.com/website/story/opinion‐you‐thought‐covid‐was‐a‐threat‐antimicrobial‐resistance‐is‐a‐close‐second/364690 [Accessed 22 March 2021]

[gpol13049-bib-0082] Paulik, L.B. , Keenan, R.E. & Durda, J.L. (2020) The case for effective risk communication: Lessons from a global pandemic. Integrated Environmental Assessment and Management, 16(5), 552–554. 10.1002/ieam.4312 32881245 PMC7461320

[gpol13049-bib-0083] Pearce, W. (2020) Trouble in the trough: how uncertainties were downplayed in the UK’s science advice on Covid‐19. Humanities and Social Sciences Communications, 7(1), 1–6. 10.1057/s41599-020-00612-w

[gpol13049-bib-0084] Pegram, T. & Kreienkamp, J. (2020) COVID‐19 and the Future of Global Health Governance: Building Back Better?. London: University College London. Available at: https://www.ucl.ac.uk/global‐governance/news/2020/nov/covid‐19‐and‐future‐global‐health‐governance‐building‐back‐better [Accessed 1 April 2021]

[gpol13049-bib-0085] Pulla, P. (2020) India expands use of controversial drug for coronavirus despite safety concerns. Nature. 10.1038/d41586-020-01619-8 32504017

[gpol13049-bib-0086] Razai, M.S. , Kankam, H.K.N. , Majeed, A. , Esmail, A. & Williams, D.R. (2021) Mitigating ethnic disparities in covid‐19 and beyond. BMJ, 372, m4921. 10.1136/bmj.m4921 33446485

[gpol13049-bib-0088] Relman, D.A. (2020) To stop the next pandemic, we need to unravel the origins of COVID‐19. Proceedings of the National Academy of Sciences of the USA, 117(47), 29246–29248. 10.1073/pnas.2021133117 33144498 PMC7703598

[gpol13049-bib-0089] Rezasoltani, S. , Yadegar, A. , Hatami, B. , Asadzadeh Aghdaei, H. & Zali, M.R. (2020) Antimicrobial resistance as a hidden menace lurking behind the COVID‐19 outbreak: the global impacts of too much hygiene on AMR. Frontiers in Microbiology, 11, 3097. 10.3389/fmicb.2020.590683 PMC7769770 33384670

[gpol13049-bib-0090] Robinson, T.P. , Bu, D.P. , Carrique‐Mas, J. , Fèvre, E.M. , Gilbert, M. , Grace, D. et al. (2016) Antibiotic resistance is the quintessential One Health issue. Transactions of The Royal Society of Tropical Medicine and Hygiene, 110(7), 377–380. 10.1093/trstmh/trw048 27475987 PMC4975175

[gpol13049-bib-0091] Rodríguez‐Verdugo, A. , Lozano‐Huntelman, N. , Cruz‐Loya, M. , Savage, V. & Yeh, P. (2020) Compounding effects of climate warming and antibiotic resistance. iScience, 23(4), 101024. 10.1016/j.isci.2020.101024 32299057 PMC7160571

[gpol13049-bib-0093] Rousham, E.K. , Unicomb, L. & Islam, M.A. (2018) Human, animal and environmental contributors to antibiotic resistance in low‐resource settings: Integrating behavioural, epidemiological and One Health approaches. Royal Society Publishing. Proceedings of the Royal Society B: Biological Sciences, 285(1876), 20180332. 10.1098/rspb.2018.0332 PMC5904322 29643217

[gpol13049-bib-0094] Ruckert, A. , Zinszer, K. , Zarowsky, C. , Labonté, R. & Carabin, H. (2020) What role for One Health in the COVID‐19 pandemic? Canadian Journal of Public Health, 111(5), 641–644. 10.17269/s41997-020-00409-z 32909226 PMC7480204

[gpol13049-bib-0095] Safitri, Y. , Ningsih, R.D. , Agustianingsih, D.P. , Sukhwani, V. , Kato, A. & Shaw, R. (2021) COVID‐19 Impact on SDGs and the Fiscal Measures: Case of Indonesia. International Journal of Environmental Research and Public Health, 18(6), 2911. 10.3390/ijerph18062911 33809128 PMC7998535

[gpol13049-bib-0096] Sager, F. , Mavrot, C. , Hinterleitner, M. , Kaufmann, D. , Grosjean, M. & Stocker, T.F. (2020) Utilization‐focused scientific policy advice: a six‐point checklist. Climate Policy, 20(10), 1336–1343. 10.1080/14693062.2020.1757399

[gpol13049-bib-0097] Sellnow, T.L. , Ulmer, R.R. , Seeger, M.W. & Littlefield, R. (2009) Effective risk communication. New York: Springer, New York.

[gpol13049-bib-0098] Shah, H. (2020) Global problems need social science. Nature, 577(7790), 295. 10.1038/d41586-020-00064-x 31942060

[gpol13049-bib-0099] Stark, A. (2018) Public inquiries, policy learning, and the threat of future crises. Oxford: Oxford University Press. 10.1093/oso/9780198831990.001.0001

[gpol13049-bib-0100] Strathdee, S.A. , Davies, S.C. & Marcelin, J.R. (2020) Confronting antimicrobial resistance beyond the COVID‐19 pandemic and the 2020 US election. The Lancet, 396(10257), 1050–1053. 10.1016/S0140-6736(20)32063-8 PMC7524538 33007218

[gpol13049-bib-0101] The Lancet Public Health . (2020) Will the COVID‐19 pandemic threaten the SDGs? The Lancet Public Health, 5(9), e460. 10.1016/S2468-2667(20)30189-4 32888438 PMC7462553

[gpol13049-bib-0102] United Nations . (2020) UN/DESA Policy Brief #81: Impact of COVID‐19 on SDG progress: a statistical perspective. Available at: https://www.un.org/development/desa/dpad/publication/un‐desa‐policy‐brief‐81‐impact‐of‐covid‐19‐on‐sdg‐progress‐a‐statistical‐perspective/ [Accessed 15 March 2021]

[gpol13049-bib-0104] Vedadhir, A.A. , Rodrigues, C. & Lambert, H. (2020) Social science research contributions to antimicrobial resistance: Protocol for a scoping review. Systematic Reviews, 9(1), 24. 10.1186/s13643-020-1279-y 32024549 PMC7003437

[gpol13049-bib-0105] Wang, J. , Mao, D. , Mu, Q. & Luo, Y.I. (2015) ‘Fate and proliferation of typical antibiotic resistance genes in five full‐scale pharmaceutical wastewater treatment plants. Science of the Total Environment, 526, 366–373. 10.1016/j.scitotenv.2015.05.046 25991498

[gpol13049-bib-0106] Weible, C.M. , Nohrstedt, D. , Cairney, P. , Carter, D.P. , Crow, D.A. , Durnová, A.P. et al. (2020) COVID‐19 and the policy sciences: initial reactions and perspectives. Policy Sciences, 53(2), 225–241. 10.1007/s11077-020-09381-4 PMC7165254 32313308

[gpol13049-bib-0107] Wellcome . (2020) The Global Response to AMR. Available at: https://wellcome.org/reports/global‐response‐amr‐momentum‐success‐and‐critical‐gaps [Accessed 22 March 2021]

[gpol13049-bib-0108] West, A.M. , Teska, P.J. , Lineback, C.B. & Oliver, H.F. (2018) Strain, disinfectant, concentration, and contact time quantitatively impact disinfectant efficacy. Antimicrobial Resistance & Infection Control, 7(1), 49. 10.1186/s13756-018-0340-2 29636911 PMC5883281

[gpol13049-bib-0109] WHO (2020) Antibiotic resistance. Available at: https://www.who.int/news‐room/fact‐sheets/detail/antibiotic‐resistance [Accessed 18 February 2021]

[gpol13049-bib-0110] WHO . (2021a) 4th Access to COVID‐19 Tools (ACT) Accelerator Facilitation Council meeting. Available at: https://www.who.int/news‐room/events/detail/2021/02/09/default‐calendar/4th‐access‐to‐covid‐19‐tools‐(act)‐accelerator‐facilitation‐council‐meeting [Accessed 18 February 2021]

[gpol13049-bib-0111] WHO (2021b) ACT‐accelerator FAQ. Available at: https://www.who.int/initiatives/act‐accelerator/faq [Accessed 18 February 2021]

[gpol13049-bib-0112] WHO . (2021c) COVAX. Available at: https://www.who.int/initiatives/act‐accelerator/covax [Accessed 18 February 2021]

[gpol13049-bib-0113] WHO . (2021d) COVID‐19 Clinical management: living guidance. Available at: https://www.who.int/publications/i/item/WHO‐2019‐nCoV‐clinical‐2021‐1 [Accessed 22 March 2021]

[gpol13049-bib-0114] WHO . (2021e) Disease outbreaks by year, WHO. Geneva, Switzerland: World Health Organization. Available at: http://www.who.int/csr/don/archive/year/en/ [Accessed 19 March 2021]

[gpol13049-bib-0115] WHO . (2021f) Global leaders unite in urgent call for international pandemic treaty. Available at: https://www.who.int/news/item/30‐03‐2021‐global‐leaders‐unite‐in‐urgent‐call‐for‐international‐pandemic‐treaty [Accessed 6 April 2021]

[gpol13049-bib-0116] WHO . (2021g) Library of national action plans. Available at: https://www.who.int/teams/surveillance‐prevention‐control‐AMR/national‐action‐plan‐monitoring‐evaluation/library‐of‐national‐action‐plans [Accessed 1 April 2021]

[gpol13049-bib-0117] WHO . (2021h) The Access to COVID‐19 Tools (ACT) Accelerator. Available at: https://www.who.int/initiatives/act‐accelerator [Accessed 18 February 2021]

[gpol13049-bib-0118] Woldehanna, S. & Zimicki, S. (2015) An expanded One Health model: integrating social science and One Health to inform study of the human–animal interface. Social Science & Medicine, 129, 87–95. 10.1016/j.socscimed.2014.10.059 25464873 PMC7115783

[gpol13049-bib-0119] World Bank . (2012) People, Pathogens and our Planet. Available at: www.worldbank.org [Accessed 19 March 2021]

[gpol13049-bib-0122] World Meteorological Organization . (2021) The state of the global climate 2020, 2021. Available at: https://public.wmo.int/en/our‐mandate/climate/wmo‐statement‐state‐of‐global‐climate [Accessed 22 March 2021]

[gpol13049-bib-0123] Yang, K. (2020) Unprecedented challenges, familiar paradoxes: COVID‐19 and governance in a new normal state of risks. Public Administration Review, 80(4), 657–664. 10.1111/puar.13248 PMC7283883 32836451

[gpol13049-bib-0124] Zhang, L. , Li, H. & Chen, K. (2020) Effective risk communication for public health emergency: reflection on the COVID‐19 (2019‐nCoV) outbreak in Wuhan, China. Healthcare, 8(1), 64. 10.3390/healthcare8010064 PMC7151105 32245157

